# University of Pennsylvania, Veterinary Department

**Published:** 1899-07

**Authors:** 


### COMMENCEMENT EXERCISES.

### UNIVERSITY OF PENNSYLVANIA, VETERINARY DEPARTMENT.

THE commencement exercises of this department were held in conjunction with the other departments of the University at the American Academy of Music, Philadelphia, on Thursday, June 15, 1899, at 11 A.M. The procession formed on the College campus at 10 A.M., marching to the Academy. The exercises consisted of Invocation by Rev. Joseph May, LL.D.; hymn, "Our Father in Heaven;" this being followed by the Commencement Address, by Hampton L. Carson, Esq.; singing of the University Hymn, "Hail, Pennsylvania;" conferring degrees in course, and conferring of honorary degrees. The exercises closed by singing of the National Anthem, "America," followed by benediction.

The degree of V.M.D. was conferred upon the following: Liberty Lyon Cheney, Southbridge, Mass.; Charles Saladine Gelbert, Jr., Scranton, Pa.; Herbert Hoopes, Bynum, Md.; Moses Jacob, Honesdale, Pa.; Clinton Foster Keiter, Williamstown, Pa.; Albert George Kern, Knoxville, Tenn.; Bassett Kirby, Woodstown, N. J.; John Philip Miller, Lebanon, Pa.; Ezra Weidman Newcomer, Mt. Joy, Pa.; Oscar Seeley, Philadelphia, Pa.; Frederick Taylor, Sewickley, Pa.; Norris Lambsen Townsend, Camden, Del.

A meeting of the Alumni Association was held in the evening at Houston Hall. Among the older graduates present were: Drs. Leonard Pearson, Charles Williams, F. H. Mackie,

A. F. Schrieber, James Mecray, Edward M. Ranck, M. E. Conard, W. P. Phipps, John W. Montague, H. D. Martein, Richard R. Mehaffey, John Pearson, E. Stanton Muir, H. B. Felton, J. D. Houldsworth, C. J. Marshall, John W. Adams, S. J. J. Harger, B. F. Senseman, W. W. Martin, Joseph Megary, L. D. Horner, and Class of '99. Others present: W. Horace Hoskins, D.V.S.

The committee in charge of the annual banquet: Drs. E. M. Ranck, Charles Williams, and C. J. Marshall. Dr. F. H. Mackie, President of the Association, acting as toastmaster, called for responses from the following gentlemen: A. F. Schrieber, "Old Remedies;" E. Stanton Muir, "Automobile *versus* Horse;" W. P. Phipps, "Horse Jokes and Horse Sense;" W. Horace Hoskins, "Government Meat Inspection;" H. B. Felton, "Canine Infirmaries;" Richard R. Mahaffey, "Veterinary Branches Taught in Agricultural Colleges;" Leonard Pearson, "The Social Standing of the Veterinarian;" M. E. Conard, "Flavor Germs in Butter;" E. M. Ranck, "Antitoxins;" M. Jacobs, "Class History for '99;" A. G. Kern, "Tennessee;" John P. Miller, "Class-spread;" L. L. Cheney, "Yankee."

Election of officers for the ensuing year resulted as follows: James M. Mecray, President; H. B. Felton, Vice-President; M. Jacob, Secretary. A committee was appointed to consider the formation of a permanent Alumni Association, and to fix regular annual dues and annual meetings: Leonard Pearson, Chairman; John W. Adams, S. J. J. Harger, M. E. Conard, C. J. Marshall.

